# Peer violence typology and modality: the associations with the mental health problems in minors

**DOI:** 10.3389/fpsyg.2026.1879354

**Published:** 2026-10-01

**Authors:** Carolina Bringas-Molleda, Benito León-del-Barco, María Isabel Polo-del-Río, Julián Álvarez-Delgado, Santiago Mendo-Lázaro, Víctor López-Ramos

**Affiliations:** Department of Psychology and Anthropology, University of Extremadura, Cáceres, Spain

**Keywords:** externalizing problems, internalizing problems, mental health, minors, peer violent, victimization

## Abstract

**Introduction:**

Violent behavior among peers affects adolescents’ physical and psychological well-being. This study aims to analyze the influence that peer violence has on mental health. First, we will analyze the psychometric proprieties of two questionnaires on victimization because of violent behaviors, both in-person and online.

**Methods:**

Participants are 600 Primary Education and Compulsory Secondary Education (ESO) students of both genders aged 10 to 17 years. The evaluation instruments used are two scales created by the study’s authors referring to peer violence, both in-person (Peer Violence Questionnaire—Victim Version) and online (Peer Cyberviolence Questionnaire—Victim Version). Likewise, they were given a third instrument–Strengths and Difficulties Questionnaire, SDQ—Self-Report Version) by Goodman (1997).

**Results:**

On the one hand, the results show an adequate validity and reliability of the two newly created instruments. On the other hand, both externalizing and internalizing problems appear more likely in minors who are the target of behaviors including physical and verbal violence, social exclusion, and threats through new technologies.

**Conclusion:**

It is necessary to recognize the types and forms of peer violence. Furthermore, it has been demonstrated that victimization, both in person and online, is associated with minors’s mental health, manifesting through externalizing and internalizing problems.

## 1 Introduction

In recent years, concern about peer-perpetrated violence has increased, occurring both within and outside educational settings. The frequency of violent behavior among peers has led to the belief that such conduct is the only strategy available to children and adolescents to resolve conflicts, while society has gradually normalized this situation (Carrascosa et al., 2019). Bullying behaviors can be classified as follows: direct or overt, aimed at individuals or property; verbal, through threats or insults; and relational or indirect, through criticism in front of others with the intent to damage the victim’s image or promote social exclusion (John et al., 2018; Cava and Buelga, 2018; Ruiz-Narezo et al., 2020; Macrynikola et al., 2021; Jiménez et al., 2024; Amnistía Internacional, 2019).

Violent behaviors among peers are explained by the interaction of multiple risk factors, including biological, social, familial, school-related, and peer-group influences. Thus, learning processes occurring during socialization shape both prosocial and antisocial behavioral characteristics (Rodríguez et al., 2010). For example, inadequate parental discipline constitutes a risk context for the development of attitudes and values that may lead to aggressive behaviors in peer relationships, particularly during early adolescence.

However, this behavioral repertoire is not exclusively shaped through interpersonal relationships but also through the use of interactive media such as social media—a virtual environment increasingly used by young people, where risk-related attitudes and behaviors may also be acquired, raising concerns about its consequences (González-Moreno et al., 2020; Núñez et al., 2021). In particular, the use of information and communication technologies (ICTs) has given rise to a new form of bullying: cyberbullying, defined as peer harassment conducted through digital technologies. These behaviors are intentional and repeated, occurring via social media and other electronic means such as mobile devices, including threatening calls, text messages, the dissemination of photos and videos, rumor spreading, privacy violations, and, in more severe cases, impersonation. All of this is directed at a person who is unable to defend themselves or control the situation, resulting in increased helplessness (Ortega et al., 2016; Marín-Cortés et al., 2019; Barlett, 2019).

Characteristics of this form of peer violence include the ease of anonymity for perpetrators, rendering physical appearance irrelevant. Similarly, unlike face-to-face interactions, perpetrators cannot observe the victim’s reaction, while victims cannot exert control over the situation, increasing their sense of insecurity without the possibility of escape, despite the absence of physical contact. Moreover, these behaviors spread swiftly and may be viewed repeatedly by an indeterminate number of individuals. They do not necessarily occur within school contexts, which makes it more difficult to prevent and increases victims’ vulnerability. This dynamic also facilitates repeated offending (Mendoza, 2014; Ordóñez and Prado, 2019; Barlett, 2019).

In any case, it should be noted that both face-to-face violence and victimization through digital technologies often occur simultaneously, resulting in a higher frequency of violent behaviors, although not necessarily a greater negative impact on victims (Núñez et al., 2021). Some studies agree that, despite the increased use of ICTs and the emergence of new forms of online violence, the prevalence of face-to-face violence remains higher than that of virtual violence (Ordóñez and Prado, 2019). These findings also suggest that cyberbullying is associated with prior experiences of traditional victimization. Similarly, individuals who have not been involved in cyberviolence have typically not experienced face-to-face violence, suggesting that prior victimization increases the likelihood of involvement in cyberbullying, although evidence for the reverse relationship is lacking (García-Fernández et al., 2016; Herrera-López et al., 2017).

Numerous studies have focused on examining factors influencing the prevalence of violent behaviors, such as emotional or internalizing problems, levels of peer acceptance or rejection, and exposure to violent behaviors within the family environment (Mestre et al., 2017; León-Moreno et al., 2019). Research has also analyzed the negative consequences associated with such behaviors, including health problems, substance use, academic difficulties, post-traumatic stress symptoms, and impairments in emotional well-being. In this regard, several studies have shown that adolescents who are victims of peer aggression report higher levels of anxiety, depression, and loneliness (Luo et al., 2022; Chu et al., 2024). In extreme cases, outcomes include self-harm and suicidal behaviors, which have also been observed among perpetrators and bystanders (Seals and Young, 2003; Estévez et al., 2006; Polo et al., 2015; Rodríguez et al., 2016). Additionally, these experiences contribute to the development of behavioral or externalizing problems that may persist into adulthood (Han et al., 2025).

Despite the existence of various instruments designed to evaluate peer victimization in children and adolescents, these present significant limitations in relation to the objectives of the present study. In particular, tools such as the Juvenile Victimization Questionnaire (JVQ), which has demonstrated a high prevalence of victimization experiences among youth (García and de Paúl, 2017), or the School Victimization Scale developed by Cava and Buelga (2018), which assesses physical, verbal, and relational violence within school contexts, allow for the estimation of different forms of violence. However, they primarily focus on the school setting and do not integrate the assessment of both offline (face-to-face) and online violence. This distinction is especially relevant, as evidence suggests that both modalities may have differential impacts on victims’ mental health, in terms of both internalizing symptoms (e.g., anxiety, depression, social withdrawal) and externalizing symptoms (e.g., aggressive behavior, impulsivity). Furthermore, available instruments do not always allow for the joint analysis of different types of violence in relation to these psychological outcomes, nor are they specifically adapted to different developmental stages.

In this regard, there is a need to develop assessment tools that integrate both modalities (offline vs. online) and types of violence (physical, verbal, and social), enabling the analysis of their relative contribution to mental health problems. This would allow for the identification of victimizing behaviors that, although they may occur in school contexts, are not limited to them and may also take place sporadically in sports, leisure environments, or even within the neighborhood. Therefore, we propose the development of two differentiated questionnaires for primary and secondary education students, along with the analysis of their psychometric properties, as an essential preliminary step to ensure valid and reliable measures that allow for the testing of the proposed hypotheses.

## 2 Objectives

The present study’s main objective is to analyze the associations of peer violence, considering both its modality (offline vs. online) and its typology (physical, verbal, and social), with mental health problems among victims, distinguishing between internalizing and externalizing symptomatology.

Specifically, the study seeks to examine the predictive capacity of each modality and type of violence on these psychological outcomes, in order to identify potential differential patterns of association.

Additionally, as a preliminary and instrumental objective, this study proposes the development and validation of two assessment instruments for peer violence—adapted for primary and compulsory secondary education students—and the analysis of their psychometric properties (factor structure, internal consistency, and validity), with the aim of ensuring reliable and valid measures for examining the proposed relationships.

Hypotheses

- H1. The different dimensions of peer violence (offline and online modalities, as well as physical, verbal, and social typologies) will be significantly associated with the presence of externalizing problems in victims, such that higher levels of victimization will be related to a greater likelihood of exhibiting this type of symptomatology.
- H2. The different dimensions of peer violence (offline and online modalities, as well as physical, verbal, and social typologies) will be significantly associated with the presence of internalizing problems in victims, such that higher levels of victimization will be related to a greater likelihood of exhibiting this type of symptomatology.

## 3 Methodology

### 3.1 Participants

Participants were selected through cluster sampling by random selection in 15 educational centers in Extremadura (Spain). Participants are in 5th and 6th grades of Primary Education and all 4 grades of Compulsory Secondary Education (ESO). A 95% confidence interval and a ± 5% margin of error were considered for the final selection of the sample; as a result, a total of 600 students participated, belonging to 8 primary schools and 7 secondary schools, with ages ranging from 10 to 17 years (M = 12.49; SD = 1.949). As for gender, 285 students were female (47.4%). The primary education students totaled 307, 182 from 5th grade (59.17%) and 125 from 6th grade (40.82%). As for Compulsory Secondary Education students, there were a total of 293, distributed as follows: 1st year of ESO (*N* = 58; 19.84%), 2nd year of ESO (*N* = 60; 20.63%), 3rd year of ESO (*N* = 127; 43.25%), and 4th year of ESO (*N* = 48; 16.26%).

### 3.2 Instruments

*Peer violence questionnaire—victim version*. This is a questionnaire created by the authors for this research based on instruments published the Ombudsman report on school violence in Spain (Defensor del Pueblo, 2000; 2007), which assesses violence among children and adolescents. To this end, the items most appropriate to the research objective were selected and adapted, and new items were proposed to broaden the assessment of violent behaviors. The final instrument consisted of 20 items reflecting violent behaviors received from peers, classified in physical behaviors, verbal behaviors, threats, or social exclusion. The answers are presented as a Likert scale of four categories (1 = Never, 2 = Sometimes, 3 = Often, and 4 = Always).

*Peer cyberviolence questionnaire—victim version*. Likewise, this instrument has been created to assess the frequency of violent behaviors from peers through new technologies. In this case, we selected the items from the Peer Violence Questionnaire (victim version) that were suitable for online administration and added several new items to provide a more comprehensive assessment of these behaviors as reported by minors. In total 11 items were included, rated on a four-category ordinal scale (1 = Never, 2 = Sometimes, 3 = Often, and 4 = Always).

*Strengths and Difficulties Questionnaire*—*SDQ, Self-report Version* (Goodman, 1997). It consists of 25 items with a response format following a 3-point Likert scale (0 = No, not at all; 1 = Sometimes; 2 = Yes, always). The items are divided into 5 factors: Emotional Problems, Behavioral Problems, Peer Problems, Hyperactivity, and Prosocial Behavior. For this study, the factors have been grouped into two categories: Externalizing Problems, including subscales Behavioral Problems and Hyperactivity; and Internalizing Problems, including Emotional Problems and Peer Problems.

The reliability indexes were as follows: Total SDQ score, Cronbach’s alpha (α = 0.83), McDonald’s omega (Ω = 0.85); Internalizing Problems, Cronbach’s alpha (*α* = 0.80), McDonald’s omega (Ω = 0.80); Externalizing Problems, Cronbach’s alpha (*α* = 0.70), McDonald’s omega (Ω = 0.73).

To determine whether the scales used adequately fit our data, different goodness-of-fit indexes were analyzed. The results showed a satisfactory model fit (χ^2^ = 99.272; χ^2^/df = 2.919), with high values in incremental and comparative fit indexes (GFI = 0.983; IFI = 0.919; CFI = 0.918), as well as a TLI index close to the recommended cutoff (TLI = 0.891). Likewise, the error indices showed adequate values (RMSR = 0.050; RMSEA = 0.067), falling within ranges considered acceptable in the literature. Overall, these findings provide evidence of the instrument’s structural validity and support the adequacy of the two-factor model for use in the analyzed sample.

### 3.3 Procedure

Prior to the research, it was approved by the Comisión de Bioética y Bioseguridad (Bioethics and Biosecurity Committee) at Universidad de Extremadura (No. 197/2024). Subsequently, we contacted the educational centers in order to explain the study’s objectives and to ask for permission to fill in the questionnaires. To this purpose, since the participants are minors, we obtained the parents’ informed consent following the ethical principles of American Psychological Association (2020). The questionnaire was handed online to each group during school hours via Google Forms, with a duration of 20 min approximately, in a quiet, distraction-free setting. The participants were informed of the purpose of the research, and all agreed to participate in the study. Subsequently, we asked the participants to be honest in their responses, guaranteeing their anonymity, confidentiality of the collected data and it exclusive use for research purposes. They were also informed of their right to withdraw from the survey at any time if they wished to do so. However, all participants completed the questionnaires and were thanked for their participation.

### 3.4 Data analysis

To examine the psychometric properties of the Peer Violence and Cyberviolence Questionnaires (victim version) developed by the authors, an Exploratory Factor Analysis (EFA) was conducted for each instrument, followed by a Confirmatory Factor Analysis (CFA) to verify the factorial structure obtained. The original sample (*n* = 600) was randomly divided into two subsamples (n1 = 300 and n2 = 300). The first subsample (n1) was used to conduct the EFA, and the second (n2) served as a validation sample for the CFA. Both subsamples were equivalent in terms of age and gender. Subsequently, the reliability of each factor was assessed using Cronbach’s alpha and McDonald’s omega coefficients.

Regarding the *Strengths and Difficulties Questionnaire, SDQ—Self-Report Version* (Goodman, 1997), a confirmatory analysis was conducted to determine whether the conceptual structure of the original study fit the present data. Reliability analyses (Cronbach’s alpha and McDonald’s omega) were also performed.

In line with the main objective of the study, a binomial logistic regression analysis was conducted to examine the relationship between one or more predictor variables and a binary dependent variable (with two possible outcomes), estimating the probability of a given event occurring. To estimate the relative contribution of predictor variables and facilitate comparisons, standardized scores were used to obtain standardized coefficients and odds ratios.

*For Hypothesis 1*, the predictor variables included dimensions of peer violence according to modality (offline vs. online), and the dependent variable was externalizing problems. Following the recommendations of Goodman (1997), scores were categorized into two groups: presence of problems (> 80th percentile) and absence of problems (≤ 80th percentile). This cutoff point makes it possible to identify scores in the upper range of the SDQ normative distribution, which are indicative of elevated levels of externalizing problems (Ortuño-Sierra et al., 2022).

*For Hypothesis 2*, the predictor variables included dimensions of peer violence according to modality (offline vs. online), and the dependent variable was internalizing problems. As in Hypothesis 1, scores were categorized into two groups (presence vs. absence of problems).

A *post hoc* statistical power analysis was conducted using G*Power 3.1 (Faul et al., 2009) with a multiple regression model. Considering the smallest observed effect size (OR = 1.376), a sample size of *N* = 600, a significance level of *α* = 0.05, an *R*^2^ value of 0.15, corresponding to an effect size of *f*^2^ = 0.176, and a total of 8 predictors, the statistical power achieved was virtually 100% (1 − *β* = 0.999), substantially exceeding the conventional threshold of 0.80.

The statistical analyses were conducted using SPSS version 27.0 for PC and Free JASP.

## 4 Results

### 4.1 The psychometric properties of the peer violence and Cyberviolence

First, the psychometric properties of the *Peer Violence Questionnaire—Victim Version* were examined. To this end, an exploratory factor analysis (EFA) was conducted, supported by the sampling adequacy measure (KMO = 0.904) and Bartlett’s test of sphericity (χ^2^ = 4938.485, df = 276, *p* < 0.001). Using the Kaiser (1960) *criterion* – eigenvalues greater than 1 – and an main components analysis extraction method with Oblimin rotation a five-factor solution was obtained (see Table 1), accounting for 61% of the total variance. The first factor corresponds to verbal violence behaviors (e.g., “They insult me”). The second factor reflects physical aggression (e.g., “They push me”). The third factor includes threats and coercion (e.g., “They threaten with telling things about me to others”). The fourth factor refers to indirect or property-related violence (e.g., “They hide my belongings”). Finally, the fifth factor encompasses social exclusion behaviors (e.g., “They do not want to work with me”).

**Table 1:** Exploratory factor analysis of peer violence questionnaire—victim version.

Items	Component					Communalities
	1	2	3	4	5	
They insult me	**0.730**	−0.076	−0.036	0.135	0.116	0.671
They speak badly of me	**0.624**	0.013	0.031	0.085	0.158	0.581
They tell me to shut up	**0.591**	0.257	0.015	0.051	−0.023	0.558
They shout to me	**0.497**	0.311	0.014	0.112	0.126	0.605
They hit me (kicking, slapping, smacking…)	0.239	**0.617**	−0.119	0.073	0.148	0.596
They pinch me	0.022	**0.405**	0.017	0.351	−0.010	0.367
They push me	0.385	**0.516**	0.098	0.181	−0.040	0.691
They pull me by the hair	−0.057	**0.719**	0.130	0.002	0.052	0.603
They threaten me to do things I do not want to do (bringing money, doing homework, etcetera)	−0.111	−0.177	**0.795**	0.078	0.112	0.656
They threaten me with objects (sticks, knives, etcetera)	0.015	0.200	**0.685**	−0.089	−0.087	0.574
They threaten with telling things about me to others	0.227	−0.019	**0.566**	0.176	0.117	0.658
They threaten me to say things I do not want to say	0.104	0.022	**0.707**	0.102	0.071	0.683
They hide my belongings	0.377	−0.134	0.030	**0.394**	0.156	0.508
They break my belongings	−0.112	−0.007	0.042	**0.778**	0.133	0.664
They steal my belongings	0.015	0.003	−0.008	**0.813**	−0.028	0.657
They throw my belongings	0.131	0.062	0.089	**0.724**	−0.127	0.619
They ignore me	0.204	0.264	0.026	−0.123	**0.616**	0.607
They do not let me do activities with them	0.122	0.042	0.129	0.131	**0.542**	0.575
They do not want to work with me	0.018	−0.090	0.010	0.006	**0.846**	0.718
They do not want to sit beside me	0.152	−0.104	0.061	0.023	**0.692**	0.608

Extraction method: main components analysis.

Bold values: Factor loadings. Rotation method: oblimin.

Bold values: Factor loadings.

The reliability indices were as follows: Verbal Violence Factor, Cronbach’s alpha (*α* = 0.79), McDonald’s omega (Ω = 0.80); Physical Violence Factor, Cronbach’s alpha (*α* = 0.69), McDonald’s omega (Ω = 0.71); Threats and Coercion Factor, Cronbach’s alpha (*α* = 0.73), McDonald’s omega (Ω = 0.79); Indirect Violence or Violence Against Property Factor, Cronbach’s alpha (*α* = 0.74), McDonald’s omega (Ω = 0.74); Social Exclusion Factor, Cronbach’s alpha (*α* = 0.78), McDonald’s omega (Ω = 0.78); Total scale reliability was: Cronbach’s alpha (*α* = 0.90), McDonald’s omega (Ω = 0.91), which showed good internal consistency.

Subsequently, to confirm the five-factor structure identified in the exploratory factor analysis, a confirmatory factor analysis (CFA) of the questionnaire was conducted. The results indicated an adequate overall model fit (χ^2^ = 551.32; χ^2^/df = 3.445), with high values in the goodness-of-fit, incremental, and comparative indices (GFI = 0.979; IFI = 0.901; CFI = 0.900), while the TLI value was slightly below the most demanding recommended criteria (TLI = 0.882). Regarding the error indices, both RMSR (0.052) and RMSEA (0.069) fell within acceptable ranges. Overall, these findings support the adequacy of the five-factor model of the *Peer Violence Questionnaire—Victim Version* and provide evidence of its structural validity for use in the analyzed sample.

Similarly, an exploratory factor analysis (EFA) was conducted to validate the *Peer Cyberviolence Questionnaire—Victim Version.* The sampling adequacy measure (KMO) yielded a value of 0.880, and Bartlett’s test of sphericity was significant (χ^2^ = 2711.149, df = 55, *p* < 0.001). Using an main components analysis extraction method with Oblimin rotation, a three-factor solution was obtained, explaining 68.85% of the total variance (see Table 2).

**Table 2:** Exploratory factor analysis of peer cyberviolence questionnaire—victim version.

Items	Component			Communalities
	1	2	3	
They insult me	**0.840**	0.006	0.053	0.755
They call me names to bother or make fun of me	**0.671**	0.142	−0.002	0.549
They speak badly of me	**0.789**	−0.002	0.080	0.688
They threaten me only to frighten me	0.423	**0.563**	−0.045	0.660
They threaten me to do things I do not want to do (bringing money, doing homework, etcetera)	−0.183	**0.955**	0.017	0.809
They threaten with telling things about me to others	0.211	**0.611**	0.043	0.556
They threaten me to say things I do not want to say	0.081	**0.794**	0.115	0.783
They ignore me	0.355	−0.026	**0.496**	0.523
They do not let me participate	0.293	−0.049	**0.672**	0.691
They do not let me do activities with them	−0.164	0.060	**0.944**	0.810
They do not want to work with me	−0.006	0.082	**0.834**	0.750

Extraction method: main components analysis.

Rotation method: oblimin.

Bold values: Factor loadings.

The first factor corresponds to verbal behaviors (e.g., “They call me names to bother or make fun of me”). The second factor includes threats (e.g., “They threaten me to say things I do not want to say”). Finally, the third factor refers to social exclusion behaviors (e.g., “They do not let me participate”).

The reliability indices were as follows: Verbal Violence Factor, Cronbach’s alpha (*α* = 0.77), McDonald’s omega (Ω = 0.79); Threats Factor, Cronbach’s alpha (*α* = 0.80), McDonald’s omega (Ω = 0.81); Exclusion Factor, Cronbach’s alpha (*α* = 0.82), McDonald’s omega (Ω = 0.82); Total scale reliability was: Cronbach’s alpha (*α* = 0.88), McDonald’s omega (Ω = 0.88).

To confirm the three-factor structure identified in the exploratory factor analysis, a confirmatory factor analysis (CFA) was conducted. The results indicated an acceptable overall model fit (χ^2^ = 280.505; χ^2^/df = 6.829), with adequate values for the incremental and comparative fit indices (GFI = 0.906; IFI = 0.911; CFI = 0.911), whereas the TLI value fell slightly below the most demanding criteria (TLI = 0.880). Regarding the error indices, both RMSR (0.060) and RMSEA (0.067) were within ranges considered acceptable. Overall, these findings support the adequacy of the three-factor structure of the *Peer Cyberviolence Questionnaire—Victim Version* and provide evidence of its structural validity for use in the analyzed sample.

### 4.2 Hypotheses H1

The binomial logistic regression analysis showed a good model fit, with a Nagelkerke *R*^2^ of 0.150 (*p* < 0.001) and a non-significant Hosmer–Lemeshow test, χ^2^ = 11.332, *p* = 0.125. This analysis showed that physical and verbal violence significantly predict the likelihood of externalizing problems in victims. However, none of the victimization typologies through new technologies significantly predicted the likelihood of these problems. As shown in Table 3, individuals who experience verbal violence are 1.376 times more likely to exhibit externalizing problems, while those exposed to physical violence are 1.384 times more likely to do so.

**Table 3:** Binary Logistic Regression Analysis Results for Predicting Externalizing Problems.

Factors	B	Standard error	Wald	gl	Sig.	Exp (B)
Verbal violence	0.319	0.163	3.816	1	0.049	1.376
Threats and coercion	0.237	0.168	1.973	1	0.160	1.267
Indirect or against-property violence	0.034	0.130	0.067	1	0.795	1.034
Social exclusion	−0.016	0.159	0.010	1	0.921	0.984
Physical violence	0.325	0.131	6.181	1	0.013	1.384
Cybervictimization: social exclusion	0.030	0.155	0.038	1	0.846	1.030
Cybervictimization: verbal violence	0.062	0.159	0.154	1	0.695	1.064
Cybervictimization: threats	−0.096	0.169	0.326	1	0.568	0.908

To assess potential multicollinearity among the eight violence factors included simultaneously in the binary logistic regression model, linear regression analyses were conducted by successively introducing each of the eight predictor variables as the dependent variable and the remaining seven variables as independent variables. The VIF values ranged from 1.625 to 2.841, indicating no substantial multicollinearity among the predictor variables.

### 4.3 Hypotheses H2

Likewise, Table 4 (Nagelkerke *R*^2^ = 0.266, *p* < 0.001, and Hosmer–Lemeshow test, χ^2^ = 4.049, *p* = 0.670) shows that violence in the form of in-person social exclusion and threats through social media will result in a higher likelihood of internalizing problems. More precisely, likelihood is 2.184 in the case of social exclusion and 1.678 for cybervictimization threats.

**Table 4:** Binary Logistic Regression Analysis Results for Predicting Internalizing Problems.

Factors	B	Standard error	Wald	gl	Sig.	Exp (B)
Verbal violence	0.312	0.174	3.223	1	0.073	1.366
Threats and coercion	0.060	0.205	0.085	1	0.771	1.061
Indirect or against-property violence	−0.115	0.152	0.570	1	0.450	0.892
Social exclusion	0.781	0.176	19.629	1	<0.001	2.184
Physical violence	−0.114	0.144	0.618	1	0.432	0.893
Cybervictimization: social exclusion	−0.306	0.169	3.276	1	0.070	0.736
Cybervictimization: verbal violence	0.236	0.171	1.920	1	0.166	1.267
Cybervictimization: threats	0.517	0.222	5.444	1	0.020	1.678

## 5 Discussion and conclusions

The main objective of this study was to analyze the associations of peer violence, considering both its modality (in-person vs. online) and its typology (physical, verbal, and social) with mental health problems among victims, distinguishing between internalizing and externalizing symptomatology. The development and validation of the questionnaires designed for this purpose constitute a key contribution of the present study. The analysis of their psychometric properties yielded robust results, supporting the validity of both instruments and enabling a more precise assessment of this phenomenon.

On the one hand, the *Peer Violence Questionnaire—Victim Version* demonstrated adequate psychometric properties, showing strong internal consistency and a clear factor structure (Costello and Osborne, 2005). The exploratory factor analysis identified five distinct factors: the first refers to verbal violence behaviors; the second to physical violence behaviors; the third assesses threats and coercion; the fourth includes indirect or property-related violence; and the fifth contemplates social exclusion behaviors. This differentiation stresses the multidimensional nature of peer violence, which is expressed through various forms with specific implications (Avilés et al., 2011; Cava and Buelga, 2018).

The five-factor structure was subsequently confirmed through confirmatory factor analysis, showing fit indices within the recommended thresholds reported in the literature and providing additional evidence of construct validity and factorial stability (Hu and Bentler, 1999; Kline, 2023). Altogether, these findings support the robustness of the questionnaire and underscore its usefulness for both research and educational and clinical practice. These results also emphasize the importance of addressing peer violence from a multidimensional perspective, as assessing different forms of violence separately allows for a more accurate understanding of victims’ experiences and the design of more tailored prevention and intervention programs.

Similarly, the psychometric properties of the second instrument – the *Peer Cyberviolence Questionnaire—Victim Version* – were analyzed. The exploratory factor analysis identified three factors: verbal behaviors, threats, and social exclusion. Subsequently, confirmatory factor analysis supported this structure, showing fit indices close to recommended values, indicating that the model is consistent and potentially generalizable. Notably, the distinction between threats and social exclusion is not always observed in previous instruments, where behaviors tend to be grouped into broader or unidimensional constructs (Buelga et al., 2019; Garaigordobil, 2013) or classified according to specific aggression typologies (Palladino et al., 2015). This distinction is particularly relevant for prevention and intervention, as it allows for the identification of specific forms of digital victimization that may occur independently and affect adolescents’ psychological well-being, as suggested by longitudinal studies and meta-analyses showing that school bullying victimization is associated with increased psychological problems in adolescents (Rivara and Le Menstrel, 2016; Sampasa-Kanyinga et al., 2018).

Regarding the main objective of this study – examining the differential contribution of modality and typology of peer violence to victims’ mental health – the results indicate that students who have experienced *physical and verbal* violence from peers are more likely to present with externalizing problems. Previous research suggests that in-person victimization is associated with both internalizing and externalizing problems, and that its severity predicts greater psychological distress in children and adolescents (Montes et al., 2022; Li et al., 2025). Although cybervictimization also affects mental health, externalizing problems appear to be more strongly associated with in-person violence. These victimization experiences may favor dysfunctional cognitive processes and externalizing behaviors, increasing the likelihood that some victims later engage in aggressive behaviors toward others (Balan et al., 2022; Luo et al., 2023). These findings support the notion of a victim–aggressor cycle, in which students exposed to *physical and verbal* violence internalize negative emotions that are later expressed through aggressive behaviors.

It is worth noting from the results (Table 3) that no dimension of cybervictimization predicts externalizing problems, which underscores the importance of the form of violence. In this regard, unlike physical and verbal violence—where direct confrontation fosters reactive responses and the externalization of frustration (Luo et al., 2023)—the digital environment imposes different dynamics. Cybervictimization is characterized by the potential anonymity of the perpetrator, the absence of physical contact, and constant exposure to a diffuse audience (Sticca and Perren, 2013). These characteristics deprive the victim of an immediate physical target against which to react and generate a profound sense of helplessness and lack of control (Runions, 2013). Consequently, the distress resulting from cyberbullying tends to be internalized, manifesting predominantly as internalizing problems—such as depressive ideation or anxiety—rather than as disruptive or aggressive behaviors toward others (Kowalski et al., 2014; Zych et al., 2015).

Our findings also indicate that individuals who experience *in-person social exclusion and threats through social media* are more likely to present with internalizing problems, with *in-person social exclusion* emerging as the modality and typology showing the strongest association with youth mental health. The effects of digital victimization on mental health are not always consistent; some studies indicate that victims of cyberbullying show higher levels of internalizing problems compared to their non-victimized peers (Molero et al., 2025; Patchin and Hinduja, 2006; Ybarra et al., 2006). However, more recent research suggests that this modality may also be associated with both internalizing and externalizing difficulties (Wang et al., 2023; Xu et al., 2024).

In conclusion, the results highlight the importance of distinguishing between different modalities and types of peer violence, as this allows for the design of more targeted and effective interventions tailored to the nature of each behavior (e.g., strategies aimed at preventing verbal aggression or threats may differ from those targeting social exclusion). Furthermore, the findings indicate that certain forms of victimization are associated with a higher likelihood of presenting with internalizing and externalizing problems in minors, underscoring the need for preventive measures and interventions that integrate both school and digital environments.

Overall, these findings underscore the importance of addressing peer violence from a multidimensional perspective, providing insight into how different modalities and types of violence relate to youth mental health (internalizing and externalizing problems). This may guide future research and inform the development of prevention and intervention strategies in educational and social contexts.

## 6 Limitations

We would like to point out that this research had some limitations, the most significant being the use of self-reports as the data collection method. Regarding self-reports for assessing peer violence and mental health problems, this measure is subject to the students’ momentary and subjective perception. Besides, mental health variables regarding internalizing and externalizing problems are extremely complex and influenced by personal, social, and contextual factors. Other limitations stem from the cross-sectional design, which makes it difficult to establish stronger inferences about the relationships between the study variables. Finally, given that both evaluation questionnaires were specifically developed for this study, it would be ideal to replicate the study with a larger sample, representative both at national and international scale, while also evaluating their psychometric properties. This would allow for an assessment of the generalizability of the findings across different contexts, thereby providing valuable insights for future research.

## Data Availability

The original contributions presented in the study are included in the article/supplementary material, further inquiries can be directed to the corresponding author.
